# Patient autonomy in the era of the sustainability crisis

**DOI:** 10.1007/s11019-024-10214-x

**Published:** 2024-06-08

**Authors:** Szilárd Dávid Kovács

**Affiliations:** Institute of Behavioural Sciences, Semmelweis University, Budapest, 1089 Hungary

**Keywords:** Climate change, Green bioethics, Patient autonomy, Patient empowerment, Sustainability

## Abstract

In the realm of medical ethics, the foundational principle of respecting patient autonomy holds significant importance, often emerging as a central concern in numerous ethically complex cases, as authorizing medical assistance in dying or healthy limb amputation on patient request. Even though advocates for either alternative regularly utilize prima facie principles to resolve ethical dilemmas, the interplay between these principles is often the core of the theoretical frameworks. As the ramifications of the sustainability crisis become increasingly evident, there is a growing need to integrate awareness for sustainability into medical decision-making, thus reintroducing potential conflict with patient autonomy. The contention of this study is that the ethical standards established in the 20th century may not adequately address the challenges that have arisen in the 21st century. The author suggests an advanced perception of patient autonomy that prioritizes fostering patients’ knowledge, self-awareness, and sense of responsibility, going beyond a sole focus on their intrinsic values. Empowering patients could serve as a tool to align patient autonomy, beneficence, and the aim to reduce resource consumption.

## Patient autonomy in contemporary medical ethics

Respect for patient autonomy is a fundamental principle in contemporary medical ethics. At its essence, patient autonomy acknowledges the unique perspectives, values, and preferences of each patient, advocating for their agency in shaping treatment plans, consenting to procedures, and navigating health care choices. (Kovács, József 2006; Beauchamp and Childress 2019) Respect for patient autonomy is included in the Declaration of Geneva since 2017, and oath takers commit to it among other professional obligations medical practitioners hold toward their patients, the profession, humankind, or themselves (Wiesing 2020). Recent medical ethical debates most commonly do not question the justification of these obligations, rather address their interaction with each other in ethically challenging situations.

Literature provides a plethora of theoretical models arguing for the ideal interaction of patient autonomy and the patient’s well-being. Cohen claims that this interaction is possible in three ways: Both obligations exist individually, thereby ranking them is required to resolve potential conflicts (discrete model); Both obligations may be expressed in different ways to conform each other (semi-discrete model); One obligation conditions the other (non-discrete model) (Cohen 2019). Akin to the Cohen’s semi-discrete and non-discrete model (Cohen 2019), Bester articulates that the concept of well-being encompasses both the medically recommended aspects and the individual’s subjective perception of what contributes to their own well-being (Bester 2020). Scholars favor the implementation of both obligations in a shared decision-making process, albeit with differing emphases on the contribution of the patient or the medical practitioner in sharing the decision (Rubin 2014; Engelsma 2023). On the other hand, certain manifestations of paternalism also receive support in literature. Wilkinson argues that one’s values transform, therefore it could be unethical to provide an alternative that harms the patient solely based on the patient’s present set of values (Wilkinson 2023). Furthermore, Chen and Das state that the medical practitioner’s expertise places them in a position of *ontological decision architects* (Chen and Das 2022).

Continual discussion persists on the ethical dilemma of honoring patients’ requests, often focusing on the issue of medical assistance in dying (Kakuk 2007; Goodman and Houk 2022), a matter deeply embedded in the ethical landscape of medicine nearly since its origins (Merino et al. 2017). The study of Goodman and Houk mentions medical assistance in dying and healthy limb amputation as illustrative examples of the misapplication of the principle respect for patient autonomy (Goodman and Houk 2022), while Pullman voices concern for medical assistance in dying being a prompt and conclusive resolution to a rather intricate matter (Pullman 2023). Further on, the issue of medical assistance in dying has gained attention recently due to its rapidly growing popularity in Canada (Pullman 2023), and to the German Constitutional Court’s order which strives to limit commercialism in medical assistance in dying (Horn 2020). The article of Rigby and Symons concludes that the potential for resolving conflicts via ethical principles is compromised, since these prima facie principles occur in arguments both supporting and opposing interventions such as medical assistance in dying (Rigby and Symons 2023).

Additionally, Kovács aims to demonstrate the inconsistency of moral evaluation in body mutilation, as gender-affirming surgery is frequently ethically recognized, while healthy limb amputation in body integrity identity disorder patients is generally not. The study argues that the ethical dilemma in both cases revolves around gaining psycho-social benefit at the expense of carrying out irreversible body modification desired by the patient. (Kovács 2009) In conclusion, it is reasonable to infer that in spite of the rather broad consensus in contemporary medical ethics that the patients’ values are crucial in decision-making, there is an evolving perspective on patient autonomy’s specific role and its limits in ethically challenging scenarios.

### Environmental considerations in medical ethics

Abbasi et al. claim that due to the current and foreseeable effects of climate change, we can speak of a global health emergency (Abbasi et al. 2023). This statement does not seem to be an exaggeration in view of the 2023 World Health Organization report, which states that on a global scale, environmental pollution and other associated environmental hazards contribute to 24% of total fatalities (World Health Organization 2023). Adjacent to direct threats for human life due to extreme weather events (Macpherson 2014; Kirsch 2020; Auckland et al. 2022; Katzenberger et al. 2022), climate change is a danger to public health in a myriad of ways. Food insecurity related to climate change has increased the prevalence of malnutrition, and diminished water quality and floods have contributed to the spread of infections (Zielinski 2022). Further risks include adverse birth outcomes, cardiovascular diseases, cutaneous conditions, various manifestations of mental health impairment (Rocque et al. 2021), and challenges to sustainability in manufacturing and infrastructure jeopardizing the access to health care (Thiel and Richie 20222022). In addition, there is an unfairness apparent, as countries with smaller contributions to climate change shoulder a heavier load of its repercussions, including its effects on health (Auckland et al. 2022; Zielinski 2022).

Not only is public health compromised by the effects of climate change, but the health care sector is also a solid contributor to the crisis. Activity in health care challenging sustainability involves the entire resource supply chain, movement of staff and patients, the wide availability of single-use medical items, and the emission of gases utilized in anesthesia and asthma inhalers into the atmosphere (Lenzen et al. 2020a; Greene et al. 2022; A. Wilkinson and Woodcock 2022; Andersen et al. 2023). Research indicates that 3,3–8,1% of the total carbon footprint in countries member of the Organization for Economic Co-operation and Development, India and China stems from health care. Moreover, the ecological footprint of health care is on the rise. (Pichler et al. 2019; Lenzen et al. 2020b) In response to this crisis, the National Health Service in England responded with an ambitious goal, to achieve zero net carbon submission by 2040 (Cameron et al. 2021). Despite a lack of widespread awareness among the public regarding health care’s role in sustainability, studies show substantial support for initiatives driving a green transformation in medicine (Liew and Wilkinson 2017; D’Ancona et al. 2021; Cameron et al. 2021).

Due to the previously described bilateral connection between climate change and health, the focus of medical ethics is inevitably shifting towards embracing aspects linked to sustainability in resource management and decreasing the environmental load. (ten Have and Gordijn 2020; Auckland et al. 2022) Richie introduces four principles of green medical ethics, which partially deviates from the more widely recognized medical ethical worldview, as it does not focus on respecting individual’s decisions, nor beneficence. The four principles of green bioethics are the *distributive justice* of general resources before providing access to special needs, *resource conservation* via prioritizing more fundamental needs, *simplicity*, described as a reduced reliance on medical intervention, and *ethical economics* as opposed to profit-oriented health care. (Richie 2019) However, patient autonomy does emerge in Richie’s philosophy in the form of *green informed consent*, a novel process of decision-making, in which medical practitioners are educated in sustainability in health care and disclose the treatment alternatives’ environmental impacts to their patients. (Richie 2023a, b) In response ten Have and Gordijn argue that medical practitioners’ primary obligation is to serve their individual patient, while they may urge policymakers to educate the public on environmental issues related to health care (ten Have and Gordijn 2023). A manifestation of *green informed consent* is the preference for dry powder inhalers, which emit fewer greenhouse gases compared to meter-dosed inhalers, while maintaining similar efficacy (Parker 2023; Richie 2023a). Reactions to Parker’s article confirm ten Have’s and Gordijn’s argumentation, that patients’ education and motivation for green options is the duty of policymakers rather than the medical practitioners’ (ten Have and Gordijn 2023; Herlitz et al. 2023; Rieder 2023).

Although originating from a common foundation of bridging sciences and humanities, medical ethics and environmental ethics have evolved into distinct fields. Common ethical challenges discussed in medical ethics revolve around the patients’ role and professional obligations in clinical research or clinical care. On the other hand, a key issue in environmental ethics is the connection between humans and the natural world. (Lee 2017) Approaches in environmental ethics are distinguished by the moral status we assign to each element of the natural world. An *anthropocentric* approach would imply that the environment is subordinated to humanity, thereby the aim of preserving nature is to benefit ourselves. More *biocentric* approaches assign moral value to certain segments of the biosphere, while *ecocentric* approaches view the ecosystem as an entity of moral value, and *land ethic* also embraces non-living elements crucial for the ecosystem, such as soil and water. (Kovács 2008; Batavia et al. 2020; Pizza and Kelemen 2023; Lee 2017)

### Advancing our ethical principles

The prevailing role and the collective understanding of the principles respect for patient autonomy and beneficence in current medical ethics clearly indicate that the incorporation of more sustainable treatment options into planning is contingent upon the patient’s preexisting values. Resnik and Pugh argue that it is appropriate for the medical practitioner to engage in a discussion about sustainability only if the inclination towards such values were disclosed by the patient. They compare the situation to a demand for cosmetic surgery, where it would seem unprofessional for a medical practitioner opposing cosmetic interventions to initiate a moral debate, thereby not respecting the patient’s autonomy (Resnik and Pugh 2023). Furthermore, as Wiesing argues in an article reviewing green medical ethics, the Declaration of Geneva expresses that the patient’s health and well-being comes first (Wiesing 2020, 2022). This notion is also confirmed in the United Nations Educational, Scientific and Cultural Organization’s Universal Declaration on Bioethics and Human Rights, which articulates that the individual’s welfare should be prioritized over advantages for society (UNESCO 2005).

The author of this study observed that the argumentation concerning green medical ethics regularly employs ethical principles, norms, and ideas formulated in the 20th century, rooted in enlightenment and humanism. While the author does not aim to reject the historical development of philosophy, nor to reinvent our ethical norms, it would seem necessary to improve our reflections to international tendencies present in the 21st century, which might undermine previous century ideals to some degree. Firstly, as elaborated, we are in a state of a global health emergency that health care significantly contributes to, among other numerous substantial impacts of climate change (Abbasi et al. 2023; WHO 2023). Although not all ramifications of the crisis are currently understood, there is a broad consensus among researchers on the urgency and severity of the issue (Oreskes 2004). Thus, the author argues that Resnik’s and Pugh’s analogy between explaining their treatment options’ effects on sustainability to the patient and persuading the patient not to request cosmetic surgery appears to be incoherent, as esthetic changes and the risks of the cosmetic intervention affect the patient and their closest social environment, counter to the sustainability crisis’s impact for humanity (Resnik and Pugh 2023). Secondly, individual autonomy’s scope and value may be constrained in this century irrespective of our preferences. This tendency is arguably most apparent in the rise of technological improvements in accumulating and processing big data, handing governments and tech companies access to private information and tools to shape behaviors (Lake 2017; Aho and Duffield 2020). The individualistic approach is likewise challenged by environmental ethics, in which the preservation of the environment is a primary obligation, and it is widely considered to assign moral values to non-human subjects and entities. (Kovács 2008; Batavia et al. 2020; Pizza and Kelemen 2023; Lee 2017).

Despite significant challenges in the 21st century facing individual autonomy, including patient autonomy, disregarding personal freedom may be counterproductive, particularly in a vulnerable situation such as in a clinical setting, as it might erode trust, compliance, and cooperation (Jennings 2009). Moreover, literature demonstrating favorable public opinion regarding green treatment alternatives despite limited public awareness of the matter implies that involving patients in decision-making may function as a mean for embracing more sustainable treatment alternatives (Liew and Wilkinson 2017; D’Ancona et al. 2021; Cameron et al. 2021). Nonetheless, the author advocates that merely exploring the intrinsic values of patients to create individually tailored treatment plans may not be sufficient in light of global trends in the 21st century, such as the increased necessity for sustainability or the possible shift in the role of individuals in society. A feasible key for providing more sustainable health care without resorting to any form of “green paternalism” could be *empowerment*. The idea of empowering patients is based on an ongoing, dynamic and caring patient-practitioner partnership, where the patient undergoes personal transformation due to continuous education and an emerging potential of self-oversight. The emphasis during this process revolves around the patient’s rights, abilities and responsibilities. (Aujoulat et al. 2007) Therefore *empowerment* not only opposes paternalism, but also Chen’s and Das’s idea of the practitioner’s role as *ontological decision architects* (Chen and Das 2022), and differs from Richie’s *green informed consent* (Richie 2023a), as more stress is put on patients’ involvement adjacent to practitioners’ education. Although patient empowerment was originally introduced for managing chronic conditions (Howorka et al. 2000), the achievable applications exceed this purpose. Patients with an enhanced knowledge and sense of responsibility may reduce their medical ecological footprint via avoiding unnecessary visits to a medical facility (Keizer et al. 2015), avoiding overmedication (Kitano et al. 2021), accommodate lifestyle changes preventing the development or exacerbation of diseases, thus demanding fewer medical resources (Ma et al. 2020). Further on, increased trust in medical practitioners lead to enhanced compliance in wearing masks during the COVID pandemic (Mallinas et al. 2021), therefore we may hypothesize a similar result in other areas, where individual decisions impact a common good, such as opting for more sustainable alternatives. As patients in the 21st century actively seek information beyond the clinical environment (Tan and Goonawardene 2017), ten Have’s and Gordijn’s conclusion is undeniable that policymaker’s bear high responsibility to endorse sustainability in health care (ten Have and Gordijn 2023). However, medical practitioners cannot withdraw from the task to empower patients, since the patient-practitioner relationship continues to serve as a cornerstone for patients’ satisfaction, sense of empowerment, and confidence, as reported by patients who regularly seek information in various sources (Tan and Goonawardene 2017).

Once we established an ethical framework centered around autonomous patients empowered to bear responsibility for themselves and for the sustainability of our health care system, the issue of whether we adopt an anthropocentric, biocentric, ecocentric, or land ethic approach has minor practical significance, as these approaches share the common goal to oppose exploiting the environment (Kovács 2008). This goal is present in Richie’s principles for green bioethics, which are oriented towards minimizing the consumption of resources and to distribute resources fairly (Richie 2019). Whilst the most radical measures for resource conservation would imply not to prolong human life via medical technologies, Riche confirms that human life remains a prima facie value (Richie 2023b). To construe the just and sustainable share of environmental resources in medicine, Dwyer employs the unit of measurement “life expectancy per ecological footprint” (Dwyer 2009). To estimate the ethicality of a medical intervention from a perspective of sustainability in accordance with Richie’s goals outlined in the principles for green bioethics, the author suggests a modified version of Dwyer’s unit of measurement. This modified unit captures a more nuanced evaluation of the benefits of a medical intervention: Improvement in quality of life per ecological load. (Felce and Perry 1995; Dwyer 2009; Richie 2019) Even though predicting the precise outcome of each medical intervention or computing their exact ecological load in a clinical setting would be unreasonable, the suggested unit could offer guidance for decision-making in green medical ethics. Nevertheless, its implementation, even as an approximation, demands the development of educational programs and standardized informational materials on the environmental load of medical interventions, alongside methods on communicating this information to patients with respect.

The sections entitled *Patient autonomy in contemporary medical ethics* and *Environmental considerations in medical ethics* expanded upon the observation that resolving ethical dilemmas is often challenging due to the fact that our set of ethical principles may be applied in favor of multiple alternatives. E.g., authorizing medical assistance in dying is simultaneously supported by the respect for patient autonomy, and opposed by the respect for human life (Rigby and Symons 2023). Thus, scholars tend to prioritize principles, as Goodman and Houk reject the idea that the respect for patient autonomy is paramount (Goodman and Houk 2022), and to describe an ideal interplay between principles, as Resnik and Pugh contend that sustainability should contribute to clinical decision-making if the patient indicates that it conforms with their value system (Resnik and Pugh 2023). In this study the author argues for advancing patient autonomy via patient empowerment and to include the ambition for sustainability in medicine. This theoretical framework corresponds with prior philosophy that the immediately communicated values of the patient may not necessarily represent their true values nor best interest (Levy 2014), however bullying or coercing the patient is disapproved of, as the emphasis remains on transparency and honesty in a long-term educational process. To achieve a coherent interaction of ethical principles, the author asserts the potential alignment between patient empowerment and rationalizing the intervention’s impact on the quality of life and its ecological footprint. In this manner the conceptual basis of this study adheres to Cohen’s non-discrete model (Cohen 2019), as patient empowerment, impact on quality of life, and ecological awareness are not independent principles, rather shaped by one another, and commonly applied to pursue sustainable health care.

## Conclusions

The interaction between the respect for patient autonomy and other ethical obligations frequently sparks debate in medical ethics. This debate also exists in regard to the ethical consumption of limited resources. However, deriving the ethical course of action solely from ethical principles drafted in the 20th century may not be sufficient amidst the global health emergency brought about by the sustainability crisis, and other trends, which dismantle our idea of individuals’ highly regarded role in society. This study contends that advancing the respect for patient autonomy to patient empowerment may serve the aim to ethically employ sustainable options in health care, thus coordinating the patient’s autonomy, beneficence, and the aspiration to reduce the use of resources. The author believes that even though medicine represents one of several sectors contributing to humanity’s ecological footprint, it remains our duty to shoulder our portion of responsibility. Thus, this study highlights the commitment in the Declaration of Geneva to serve humanity (Wiesing 2020).
